# Effects of mastication on antibody production under fasting conditions in mice

**DOI:** 10.7150/ijms.80396

**Published:** 2023-01-22

**Authors:** Seonbu Yang, Yujun Park, Taesaeng Choi

**Affiliations:** Department of Microbiology, College of Medicine, Dankook University.

**Keywords:** fasting, chewing, leptin, corticosterone, immunization, antibody

## Abstract

Chewing is beneficial not only for digestion and absorption of food, but also for various physiological functions, such as cognition and immunity. In this study, the effect of chewing on hormonal changes and the immune response was investigated under fasting conditions in mice. We investigated leptin and corticosterone levels, which are hormones with well-known associations with immune response and large changes during fasting. To study of effects of chewing under fasting conditions, one group of mice was provided with wooden sticks to stimulate chewing, one group was supplemented with 30% glucose solution, and one group received both treatments. We examined changes in serum leptin and corticosterone levels after 1 and 2 d of fasting. Antibody production was measured 2 weeks after subcutaneous immunization with bovine serum albumin on the last day of fasting. Under fasting conditions, serum leptin levels decreased and serum corticosterone levels increased. Supplementation with 30% glucose solution during fasting increased leptin levels above normal, but had little effect on corticosterone levels. In contrast, chewing stimulation inhibited the increase in corticosterone production, but did not affect the decrease in leptin levels. Antibody production significantly increased under separate and combined treatments. Taken together, our results showed that chewing stimulation during fasting inhibited the increase in corticosterone production and improved antibody production after immunization.

## Introduction

Malnutrition decreases most physiological activities in mammals, including immune function. Thus, individuals with limited access to food are vulnerable to infection, as observed in developing countries, which also negatively affects the efficacy of vaccination 1. Most animal studies on malnutrition involve limiting the food supply or supplying water alone instead, and they have reported various hormonal changes during fasting, such as changes in leptin, insulin, neuropeptide Y, ghrelin, resistin, and corticosterone levels 2,3.

Mastication (chewing) is involved in the maintenance of cognitive functions and various physiological functions in addition to digestion and absorption of food 4. Recent studies have shown that glucagon-like peptide 1 secretion is increased by increasing the duration of chewing, which also affects satiety and weight loss to a certain extent 5. In both animal and human studies, stimuli of chewing are closely related to hippocampus-dependent cognitive function, a region of the central nervous system 6. Additionally, many studies have shown that chewing reduces stress coping behaviors and levels of the stress hormones cortisol and corticosterone under stress conditions in humans and animals 7,8.

Among the many hormones that are affected during fasting, we focused on leptin and corticosterone in this study. Although leptin was previously discovered as an appetite-suppressing hormone in early studies, further studies have shown that this hormone is also strongly involved in the activation of immune cells and that most immune cells express leptin receptors 9-11. Moreover, corticosterone induced by stress stimuli suppresses the proliferation of immune cells and induces apoptosis 12-14. Previous studies have reported decreased serum leptin levels and increased corticosterone levels during fasting 15,16.

In this study, we investigated the effects of chewing stimulation under fasting conditions on blood leptin and corticosterone levels and on antibody production after immunization. In addition, to investigate the chewing effect in more detail, another control group in which glucose was added to drinking water without chewing stimulation was evaluated in the fasting state.

## Materials and Methods

### Animal experiments

We used ICR female mice aged 7-8 weeks. The mice were maintained in a 12-hour light/12-hour dark cycle at 24 ± 1°C and 50% relative humidity. Mice were placed in a clean cage and fasted for 24 or 48 h starting at 8:00 AM. Few mice were supplemented with glucose solution (30% [w/v] glucose in tap water) instead of tap water, and another group of mice was provided wooden sticks to chew. For the latter, mice were pre-trained by allowing them to chew on bamboo wooden sticks (length: 4 cm, diameter: 5 mm) for 3-4 d before the experiment. Mice that did not chew on the sticks during this period were excluded from the experiment. Body weights were recorded at 0, 24, and 48 h after initiation of fasting and presented as percentages relative to those of normally fed mice (100%). For serum sampling, whole blood was collected from the tail vein, and the blood was allowed to clot at room temperature for 20 min followed by centrifugation for 10 min at 1,500 × g. The serum was aliquoted and kept frozen at -80°C until use. All animal studies were conducted in compliance with guidelines set forth by the Care and Use of Research Animals and were approved by the Animal Studies Committee of Dankook University (approval number: DKU-20-048).

### Leptin and corticosterone level measurements

Since daily changes in serum levels of leptin and corticosterone have been reported previously (17,18), the blood samples in this study were collected between 9:00 AM and 11:00 AM each day to reduce the occurrence of possible errors. Serum leptin and corticosterone levels were measured using the EzWay Mouse Leptin enzyme-linked immunosorbent assay (ELISA) kit (Cosmo bio, San Diego, CA, USA) and the Corticosterone Parameter Assay Kit (R&D Systems, Minneapolis, MN, USA) according to the manufacturers' instructions.

### Immunization and determination of antibody titers

Bovine serum albumin (BSA; Sigma-Aldrich, St. Louis, MO, USA) was used as an antigen. Mice were randomly assigned to five groups (n = 5-7 mice per group) and treated as follows: control (no fasting), fasting (fasting only), 30% glucose (supplementation with 30% glucose solution during fasting), chewing (provided with wooden sticks for chewing stimulation during fasting), and 30% glucose and chewing (supplementation with 30% glucose solution and proved with wooden sticks during fasting). The mice from each group were immunized subcutaneously with BSA (5 µg/100 µL Dulbecco's phosphate-buffered saline solution) at the end of the fasting period. At 2 weeks after immunization, blood was collected from the tail vein to measure serum levels of the BSA-specific antibody. The antibody titer was measured via indirect ELISA, which was performed in 96-well polystyrene plates. The plates were coated with 100 µL of 20 µg/mL BSA in 0.05 M carbonate-bicarbonate buffer (pH 9.6) and incubated overnight at 4°C. Unbound antigen was removed by washing three times with phosphate-buffered saline (PBS) containing Tween-20 (PBS-T; 20 mM PBS, pH 7.4, and 0.05% Tween-20), and the unoccupied binding sites were blocked by adding 2% nonfat skim milk in PBS-T and incubating the plates for 1 h at room temperature. After incubation with mouse sera for 1 h at room temperature, the plates were washed three times with PBS-T. Bound antibodies were quantified using anti-mouse horseradish peroxidase-conjugated immunoglobulin G antibodies (Santa Cruz Biotechnology, Dallas, TX, USA). BSA-specific antibody levels in the serum were determined by measuring the absorbance at 450 nm using a spectrophotometer (Bio-Rad, Hercules, CA, USA) and represented as the mean endpoint titers: the highest serum dilution levels that produced absorbance exceeding 0.1.

### Statistical analysis

Statistical analyses were performed using GraphPad Prism 8.01 (GraphPad, San Diego, CA, USA). Significant differences among groups were assessed using one-way ANOVA analysis of variance. Results are presented as the mean ± standard error of the mean, and p-values are presented as follows: *p < 0.05; ** p < 0.01; and *** p < 0.001.

## Results

### Serum levels of leptin and corticosterone under fasting conditions and antibody production after immunization

**Figure 1:**
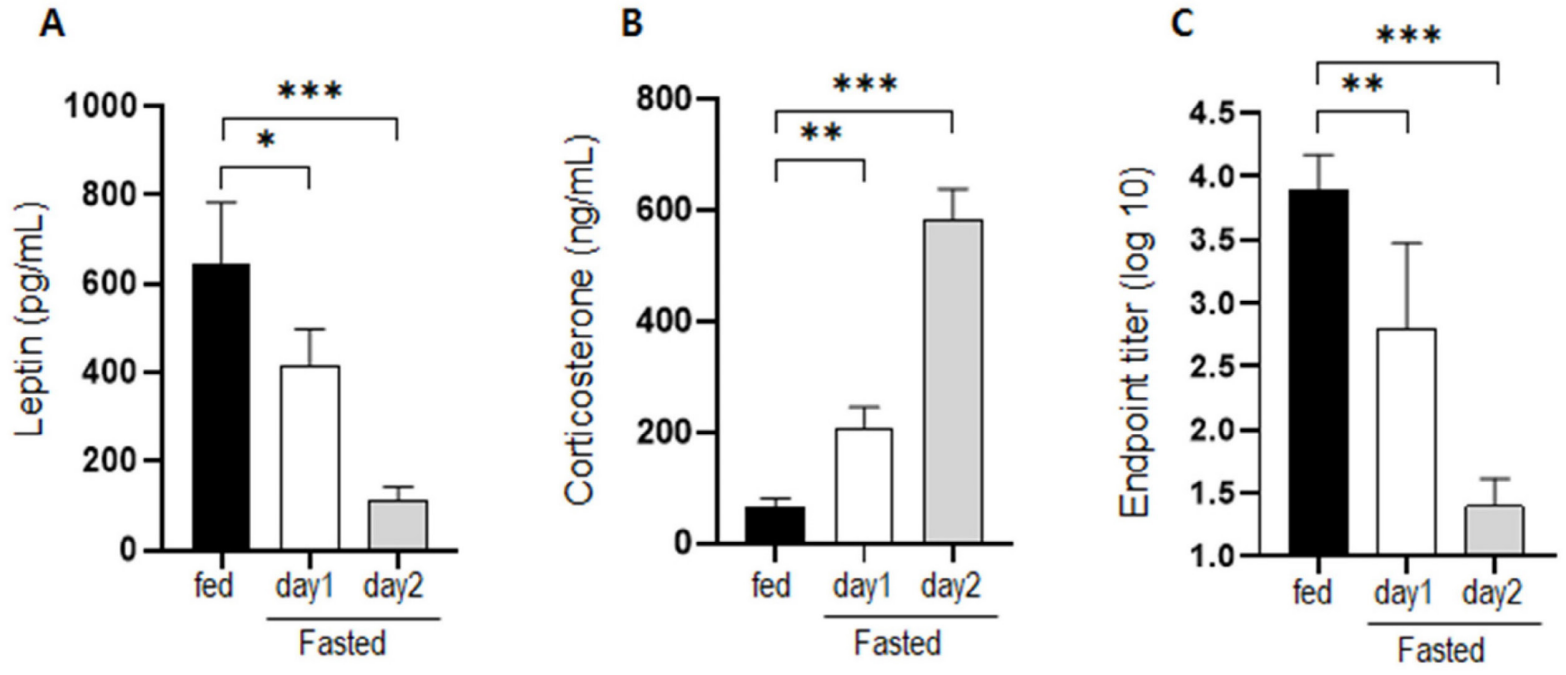
** Changes in hormone levels and antibody production after fasting.** Serum (a) leptin and (b) corticosterone levels were measured in normally fed and fasting (1 d and 2 d) mice. The levels observed for the control mice were considered the baseline level (100%), and levels in the fasting mice were normalized relative to the baseline. (c) Mice were immunized after fasting for 1 or 2 d, and antibody production was evaluated 2 weeks later. The values are presented as the mean ± standard deviation of end point enzyme-linked immunosorbent assay antibody titers of 5-6 mice per group. The data are presented as the mean ± standard deviation of five mice per group. Different superscript letters represent significantly different values (* p < 0.05, ** p < 0.01, *** p < 0.001).

Changes in the serum levels of leptin and corticosterone were measured on days 1 and 2 of fasting. Leptin levels decreased significantly in mice that had fasted for 1 and 2 d (Figure 1a). Corticosterone levels increased significantly by approximately 3-fold after fasting for 1 d and by approximately 9-fold after fasting for 2 d (Figure 1b). The level of BSA-specific antibody in mice that were supplied with normal food and mice that fasted for 1 or 2 d was measured at 2 weeks after immunization. Antibody production in fasting mice was significantly inhibited compared to that in normally fed mice (Figure 1c).

### Effects of chewing stimulation and glucose supplementation on serum levels of leptin and corticosterone and body weight in fasting mice

**Figure 2:**
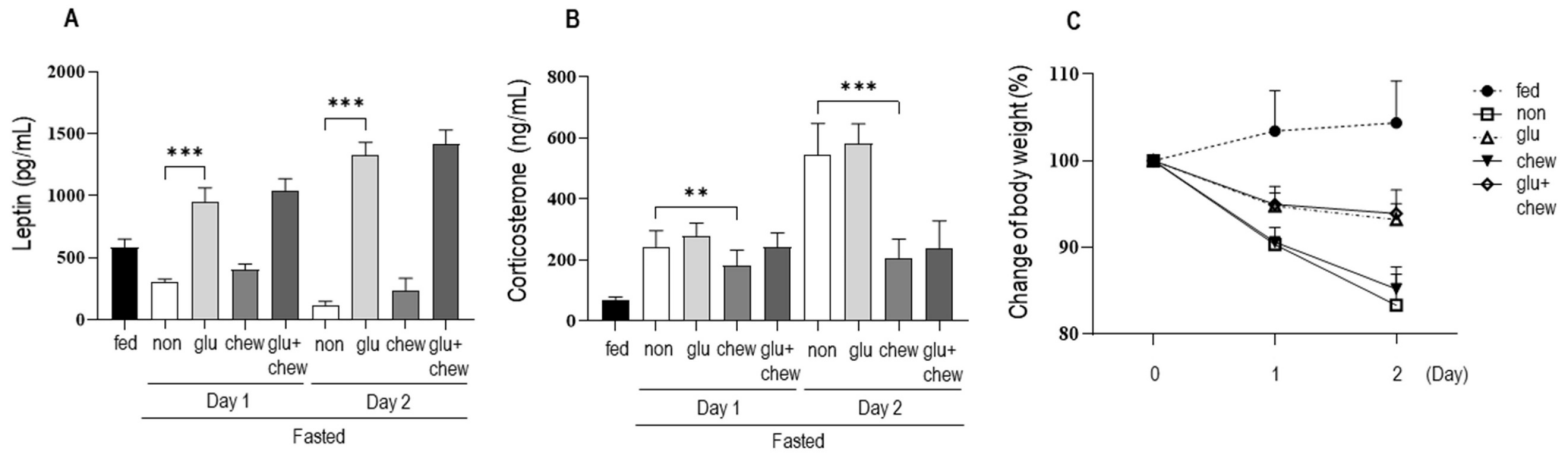
** Changes in serum hormone levels and body weight following supplementation with glucose and wooden stick during fasting conditions. a)** Leptin and** (b)** corticosterone levels were measured in mice subjected to 30% glucose supplementation, chewing stimulation, and both treatments. **(c)** Changes in body weight for each group are presented as percentages. Mice were assigned to four groups: control, non, Glu, Chew and Glu+Chew. Control, normally fed mice; non, fasting mice; Glu, 30% glucose-supplemented mice; Chew, mice provided wooden stick; Glu+Chew, mice supplemented with 30% glucose and a wooden stick.

We measured changes in the serum levels of leptin and corticosterone and body weight in mice supplemented with glucose solution and/or subjected to chewing stimulation (Figure 2). Leptin levels, which decreased significantly under fasting conditions, increased on days 1 and 2 in mice supplemented with glucose solution compared to that in the fasting group. Moreover, there was no significant change in leptin levels in mice that were provided wooden sticks (Figure 2a). Corticosterone levels in mice supplemented with glucose solution under fasting conditions did not differ significantly from the level in control mice under fasting conditions (Figure 2b). However, chewing stimulation inhibited the increase in corticosterone level induced by fasting. In the group that was provided both glucose and a wooden stick, blood leptin levels were high, which was similar to that in the group administered with glucose alone. In addition, corticosterone level was low, which was similar to that in the group provided with the wooden stick alone.

Under fasting conditions, the body weight decreased to 90% on day 1 and 83% on day 2. The body weights of mice that were supplemented with glucose decreased to 95% on day 1 and to 93% on day 2 of fasting. The body weights of mice subjected to chewing stimulation decreased to 91% on day 1 and 85% on day 2, similar to the body weights of mice under fasting conditions (Figure 2c). Thus, chewing stimulation did not affect fasting-induced weight loss, and when glucose was provided, the weight loss caused by fasting was suppressed by approximately 50%.

### Effect of chewing stimulation or glucose supplementation under fasting condition on the antibody production after immunization

**Figure 3:**
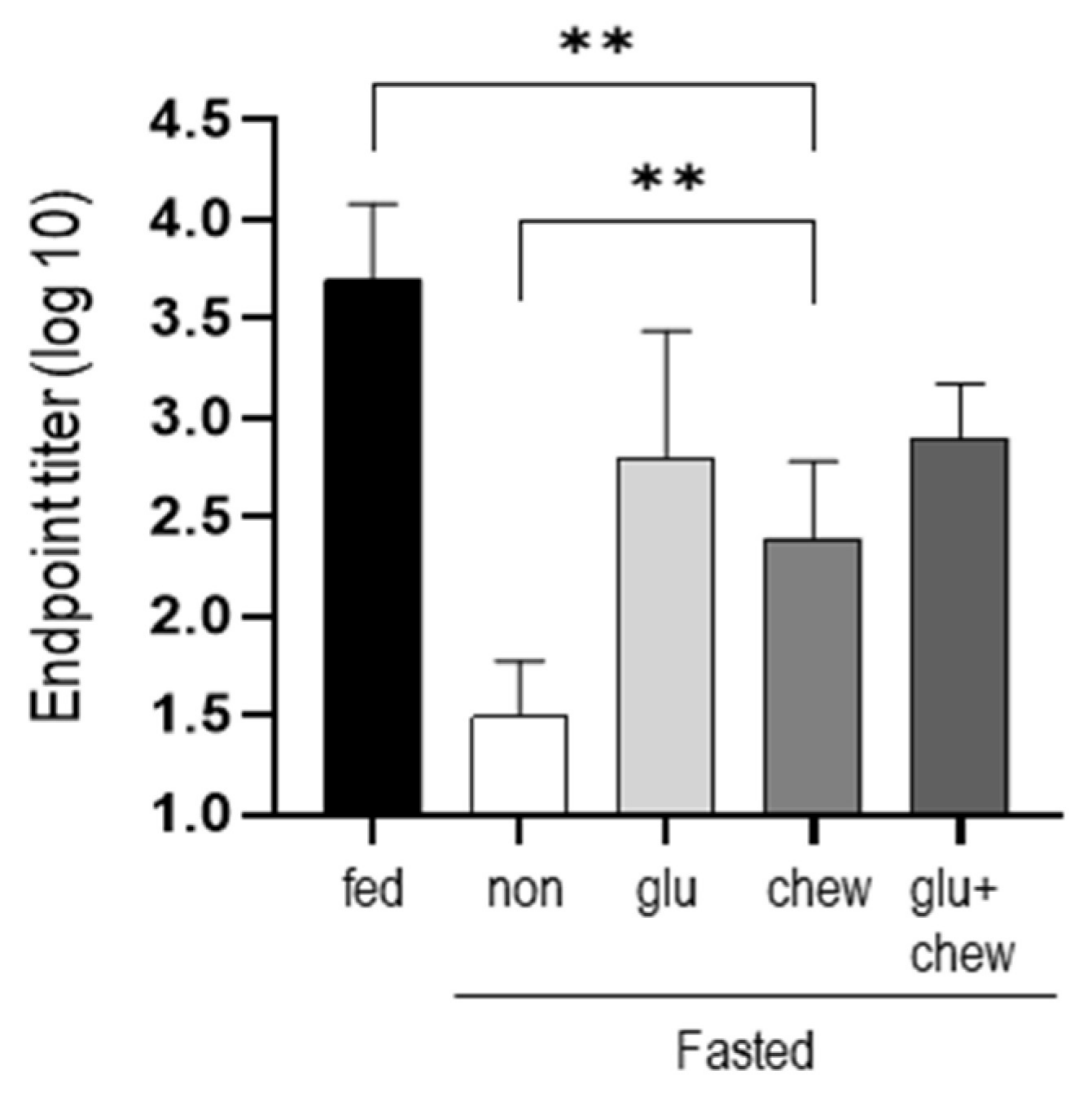
** Antibody titers were measured at 2 weeks after immunization on day 2 of fasting.** After fasting for 2 d, mice subjected to 30% glucose supplementation, chewing stimulation, and both treatments were immunized, and the serum antibody levels were measured using ELISA at 2 weeks after immunization. The values are presented as the mean ± standard deviation of end point ELISA antibody titers of 5-6 mice per group. Fed, normally fed mice; non, fasting mice; Glu, 30% glucose-supplemented mice; Chew, mice provided wooden stick; Glu+Chew, mice supplemented with 30% glucose and a wooden stick; ELISA, enzyme-linked immunosorbent assay.

Mice were fasted for 2 d in each condition and immunized with BSA. Antibody production was measured at 2 weeks after immunization in mice that had fasted for 2 d and in mice supplemented with glucose and/or provided a wooden stick during the fasting period. Antibody production was significantly higher in mice fed glucose or/and provided wooden sticks compared to that in mice that fasted, although the level did not reach that in mice fed a normal diet (Figure 3).

## Discussion

Chewing has been shown to affect various physiological and immune response. Therefore, we investigated the effects of chewing stimulation under fasting conditions on blood leptin and corticosterone levels and on antibody production after immunization. Blood leptin levels decreased rapidly after fasting, while the corticosterone level in the blood increased sharply. The decrease in leptin level was attributed to its secretion *in vivo* as an appetite-suppressing hormone secreted from the adipose tissue upon food intake 19, 20. In addition, the increase in level of corticosterone secreted during the stress response was attributed to the extreme stress of fasting 21,22. Moreover, the results of the subcutaneous immunization test of BSA on the second day of fasting showed a low level of antibody production compared to that in mice fed a normal diet, which was similar to that reported previously 23.

One of the causes of the decrease in antibody production under fasting conditions may be the low level of leptin. Leptin is involved in the immune response and survival, proliferation, and activity of immune cells, and consequently plays an important role in humoral immunity as reported in studies using ob/ob mice genetically lacking leptin 23-26.

Stress conditions negatively affect immune responses, and corticosterone/cortisol, a representative stress hormone, is involved in this phenomenon 27,28. Therefore, although other effects due to fasting cannot be excluded, low leptin levels and high corticosterone levels under fasting conditions may be strongly involved in low humoral immunity.

In this study, we found that when the chewing stimulation was induced by providing a wooden stick under fasting conditions, there was no significant change in the leptin level; however, the increase in corticosterone level was significantly suppressed. This is consistent with the results of previous studies in which increase in stress hormone production is inhibited by chewing stimulation 29,30. Regarding this mechanism, it has been reported that stimulation by mastication inhibits the release of corticotropin-releasing factor in the hypothalamus or directly or indirectly suppresses the stress response 31. In contrast, mice that were provided glucose had a higher leptin level than that in control mice, whereas there was a minor change in the increase of corticosterone production. Notably, when glucose was provided, the increase in corticosterone production was not inhibited at all (Fig. 2b). It cannot be assumed that drinking water containing glucose provided the same level of nutrients as in the case of free feeding. However, based on the results of weight change (Fig. 2c), glucose supplementation had an inhibitory effect on weight loss of approximately 50%. If it is assumed that the stress provided to mice under fasting conditions is divided into the absence of food intake and the absence of mastication (chewing), these results suggest that the stress associated with the increase in blood corticosterone level is more significantly related to the absence of chewing.

Under fasting conditions, blood leptin level was low. In contrast, leptin level was significantly higher in the group in which glucose was added to drinking water under fasting conditions than that in the control group (Fig. 2b). This is probably related to the induction of leptin expression by increased glucose levels in the blood 32,33. Nocturnal rodents have elevated leptin levels several hours after food intake at night 34. Thereafter, the leptin level gradually decreases. In contrast, if glucose is provided in drinking water and if the mouse frequently ingests water regardless of day or night, the higher leptin level in the group receiving glucose-containing water than that in the control group is reasonable.

In this study, antibody production was evaluated as an index of recovery of immunity, and this was investigated under additional conditions during fasting. Antibody production, which was suppressed under fasting conditions, was significantly restored in both cases when glucose was supplied and when wooden sticks were provided. When both glucose and wooden sticks were provided together, no synergistic effect was observed. Recently, Deng et al. reported that antibody production under fasting conditions in mice is recovered by administering recombinant leptin 23. These results indicate that antibody production is closely related to blood leptin concentration. However, our study showed that despite low leptin concentrations, antibody production was improved in fasting mice when chewing suppressed the increase in corticosterone production. We believe that the effect of mastication cannot be limited to suppression of increased corticosterone production. Many studies on the effect of mastication have been conducted under more diverse conditions such as soft-diet feeding, molar extraction, or bite-raising 35. In this study, the effect of chewing on a wooden stick provided under the condition of fasting, which is a very stressful situation, was investigated. Therefore, it is difficult to generalize this result of this study. However, the interesting part of this study is that the ability to produce antibodies can be restored to some extent by chewing despite low leptin levels induced by fasting.

In conclusion, we confirmed that chewing stimulation induced by wooden sticks under fasting conditions in mice suppressed the increase in corticosterone production and also enhanced antibody production.

## Abbreviations

ELISA: enzyme-linked immunosorbent assay
BSA: bovine serum albumin
